# Self-triggered assistive stimulus training improves step initiation in persons with Parkinson’s disease

**DOI:** 10.1186/1743-0003-10-11

**Published:** 2013-01-30

**Authors:** Robert A Creath, Michelle Prettyman, Lisa Shulman, Marjorie Hilliard, Katherine Martinez, Colum D MacKinnon, Marie-Laure Mille, Tanya Simuni, Jane Zhang, Mark W Rogers

**Affiliations:** 1 Department of Physical Therapy & Rehabilitation Science, University of Maryland School of Medicine, 100 Penn Street, Room 115, Baltimore, MD, 21201, USA; 2 Department of Neurology, University of Maryland School of Medicine, Baltimore, MD, USA; 3 Northwestern University Feinberg School of Medicine, Department of Physical Therapy & Human Movement Sciences, Northwestern University, Chicago, IL, USA; 4 Department of Neurology, Northwestern University Feinberg School of Medicine, Chicago, IL, USA

**Keywords:** Step, Initiation, Parkinson’s, Freezing, Hesitation, Intervention

## Abstract

**Background:**

Prior studies demonstrated that hesitation-prone persons with Parkinson’s disease (PDs) acutely improve step initiation using a novel self-triggered stimulus that enhances lateral weight shift prior to step onset. PDs showed reduced anticipatory postural adjustment (APA) durations, earlier step onsets, and faster 1^st^ step speed immediately following stimulus exposure.

**Objective:**

This study investigated the effects of long-term stimulus exposure.

**Methods:**

Two groups of hesitation-prone subjects with Parkinson’s disease (PD) participated in a 6-week step-initiation training program involving one of two stimulus conditions: 1) Drop. The stance-side support surface was lowered quickly (1.5 cm); 2) Vibration. A short vibration (100 ms) was applied beneath the stance-side support surface. Stimuli were self-triggered by a 5% reduction in vertical force under the stance foot during the APA. Testing was at baseline, immediately post-training, and 6 weeks post-training. Measurements included timing and magnitude of ground reaction forces, and step speed and length.

**Results:**

Both groups improved their APA force modulation after training. Contrary to previous results, neither group showed reduced APA durations or earlier step onset times. The vibration group showed 55% increase in step speed and a 39% increase in step length which were retained 6 weeks post-training. The drop group showed no stepping-performance improvements.

**Conclusions:**

The acute sensitivity to the quickness-enhancing effects of stimulus exposure demonstrated in previous studies was supplanted by improved force modulation following prolonged stimulus exposure. The results suggest a potential approach to reduce the severity of start hesitation in PDs, but further study is needed to understand the relationship between short- and long-term effects of stimulus exposure.

## Background

Of the many debilitating symptoms present in Parkinson’s disease (PD), hesitation associated with freezing of gait (FOG) is a common manifestation of the disease. Approximately one third of individuals with PD experience transient breaks in voluntary motor activity that interfere with executing complex movements or switching between different movements [1].

During gait initiation, an anticipatory postural adjustment (APA) phase precedes stepping [2-6]. For forward stepping, these APAs involve muscle-activated changes in ground reaction forces that shift the center of pressure backward and toward the initial swing limb, propelling the body center of mass forward and towards the single-stance limb prior to stepping. Compared to healthy subjects, the medio-lateral (M-L) and antero-posterior (A-P) ground forces and center of foot pressure changes characterizing APAs in PD patients are longer in duration and reduced in amplitude with prolonged delays between APA onset and step onset [7-10]. Moreover, while APAs are normally present during voluntary step initiation, they are often absent in PD patients experiencing hesitation delays [4,9].

A longstanding clinical observation indicates that difficulties with initiating locomotor movements such as gait with PD may be transiently overcome if the normally automatic APAs that precede and accompany such movements (I.e. lateral weight shift during step initiation) receive modest manual assistance from a clinician [11]. This observation suggests a possible disruption in the normal coupling between posture and locomotion. In this regard, neurophysiological studies have indicated that the control of posture and locomotion are normally interdependent at many levels of the central nervous system (CNS) involving supraspinal and spinal networks [12-15]. What remains to be determined, however, are the ways by which locomotion may be affected by initial postural conditions. This issue appears to have important implications for current rehabilitative interventions in PD which mainly focus on separate aspects of the problems such as posture and balance training [16] or gait training [17-19].

We have previously shown that the APA-stepping sequence in PD subjects could be improved through neuromechanical assistance with lateral weight transfer prior to step onset [20]. In these experiments we introduced a novel self-triggering stimulus loosely based on the aforementioned manual assistance during step initiation from a clinician [11]. In the case of manual assistance, the clinician provides mechanical assistance that enhances the deficient lateral weight shift observed in PD prior to stepping. In the self-triggering paradigm the stimulus is activated by the subject instead of a clinician. When a PD subject attempts step initiation, deficiencies can be overcome by a computer-controlled device that responds to their attempt at lateral weight shift by measuring changes in ground reaction forces and providing mechanical enhancement while they are shifting weight. The self-triggering paradigm requires the subject to initiate the movement, but provides real-time assistance if movement deficiencies exist, improving the coupling between postural control and stepping control during step initiation [20].

The responsiveness of hesitation-prone PD subjects to a small-magnitude, posture assistance stimulus (lateral displacement of the pelvis using a computer-controlled, motor-driven device incorporating cables attached to a waist belt) was demonstrated following a 50-trial training intervention. Subjects were tested without the stimulus before and immediately after training showing decreases in APA duration, earlier step onset times, and faster first step speeds. Furthermore, step duration was retained one-week post-training [20]. To more directly facilitate the changes in APA forces affected indirectly by the waist-pull stimulus, a drop (or elevation) of support surface beneath the stance foot was substituted for the lateral waist-pull stimulus [21]. PD subjects responded favorably to the posture assisted locomotion (PAL) drop stimulus that reinforced the intended APA action by showing reduced APA time durations, increased peak APA amplitudes, and earlier step onset times.

Because gait and balance problems often respond poorly to treatment with anti-parkinsonian medications, and to other interventions such as deep brain stimulation [22,23], physical therapy interventions are an important clinical treatment for individuals with PD [19,24,25]. For acute applications, the PAL stimulus has been useful for improving the linkage between posture and locomotion during gait initiation, but its effectiveness for long-term applications remains unknown. The purpose of this study was to assess the feasibility of a training program incorporating the PAL stimulus that could ultimately be applied clinically in order to help PD patients overcome difficulties associated with start hesitation and possibly freezing of gait. With the application of this intervention we expected to see improved APA and stepping performance consistent with reduced start hesitation.

## Methods

### Subjects

Fifteen subjects (11Male/4Female, 73.1 ± 8.5 yrs) with idiopathic PD were randomly assigned to one of the two groups (see Table 1): Drop stimulus group (n = 7) and Vibration stimulus group (n = 8). Subjects provided informed consent consistent with the policies of the Institutional Review Boards at Northwestern University School of Medicine and the University of Maryland School of Medicine, as well as the Declaration of Helsinki.

**Table 1:** Subject information

**Subject #**	**training group**	**Age**	**Gender**	**PD duration**	**H&Y**
1	drop	81	M	10	3
2	drop	58	F	23	2.5
3	drop	75	F	6	2
4	drop	70	M	6	2.5
5	drop	65	M	21	3
6	drop	65	M	9	2
7	drop	81	M	8	2.5
	ave	70.7		11.9	2.5
	std	8.7		7.1	0.4
8	vibration	77	M	9	2.5
9	vibration	78	M	1	2.5
10	vibration	68	M	4	2
11	vibration	81	F	7	3
12	vibration	87	M	5	2.5
13	vibration	69	M	3	2.5
14	vibration	62	F	4	3
15	vibration	80	M	6	2.5
	ave	75.3		4.9	2.6
	std	8.2		2.5	0.3
	All subjects				
	ave	73.1		8.1	2.5
	std	8.5		6.1	0.4
	Group comparison				
	p(alpha = .05)	0.319		0.021	0.745

Inclusion criteria included: 1) the diagnosis of adult idiopathic onset PD; 2) a history of FOG as evidenced by self-report and clinical assessment; 3) a stable regimen of anti-parkinsonian medications; 4) ability to walk at least 10 m without assistance; 5) stage 2–3 of the Hoehn and Yahr disability scale [26]; 6) a score > 24 on the Mini Mental State Examination [27].

Exclusion criteria included: 1) evidence of any clinically significant functional impairment related to cardiovascular, pulmonary, metabolic, other neurologic, or musculoskeletal disease criteria that would preclude participation in training; 2) any medical condition that might require other medical or surgical treatment during the study period; 3) a history of brain surgery or placement of a deep brain stimulator; 4) dyskinesias > grade 2 on the Unified Parkinson’s Disease Rating Scale (UPDRS); 5) any uncorrected vision or hearing problems that limit daily activities or communication.

Clinical PD motor assessments were performed by a movement disorders research coordinator. All assessments, experimental testing, and training were performed in the medications ON state, defined as the period of maximal therapeutic benefit achieved after a patient takes their usual doses.

### Apparatus

Subjects wore a safety harness that prevented falls and initially stood quietly facing along a 4.88 m (16 ft) × 1.22 m (4 ft) × 0.39 m (15.375 in.) elevated walkway. Their feet were positioned comfortably and marked on the contact surface to maintain consistent foot placement.

**Figure 1:**
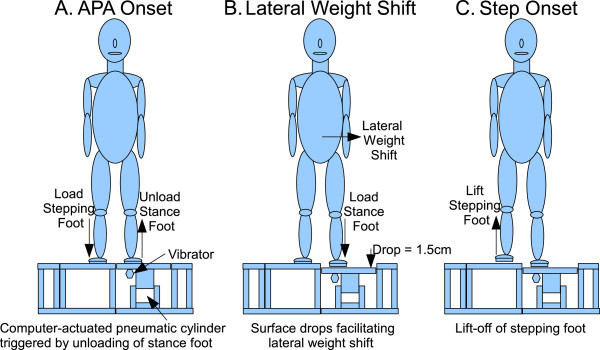
Experimental apparatus: the PAL drop stimulus during step initiation at APA onset (A), during lateral weight shift (B) and at step onset (C).

The PAL Drop training apparatus (Figure 1) consisted of a computer-controlled pneumatic piston which supported the support surface under the subject’s stance foot [21]. Once activated, the piston quickly lowered the support surface 1.5 cm in a time of approximately 100 ms. Activation was triggered immediately after APA onset when the subject actively decreased vertical force beneath the stance foot by 5% from the pre-trial baseline value (Figure 1A). The drop stimulus facilitates lateral weight shift and loading of the stance limb (Figure 1B). Once single limb support is established, step onset begins with lift off of the stepping foot (Figure 1C).

Vibration training substituted a vibratory stimulus in place of the drop stimulus that was activated using the same timing-trigger algorithm as the drop. The computer-controlled vibrator, which was affixed to the underside of the support surface directly beneath the subject’s stance foot (Figure 1A), vibrated at 200–250 Hz for 100 ms with amplitude of approximately 0.1 mm. Prior observations (unpublished) suggested that subjects may receive timing cues from the drop stimulus in addition to mechanical assistance at the onset of lateral weight shift. The purpose of the vibratory stimulus was to provide a non-mechanical stimulus, i.e. a timing signal, which controlled for this.

### Data collection

Vertical ground reaction forces were measured using two strain-gauge force platforms (AMTI, Newton, MA). Kinetic data was collected at 500 Hz. 3-D kinematic data was collected using a 6-camera Vicon data collection system at 120 Hz. Reflective markers were placed on the subject’s left and right lateral malleoli. Data collection and external triggering were computer-controlled using a custom LabVIEW program (National Instruments, Austin Texas).

### APA and stepping characteristics

APAs were assessed by measuring changes in vertical ground reaction forces. Stepping-foot APA onset occurred when the vertical force increased three standard deviations above its baseline value (subjects started each trial in quiet stance). Stance-foot APA onset occurred when the vertical force decreased three standard deviations from its initial baseline value.

**Figure 2:**
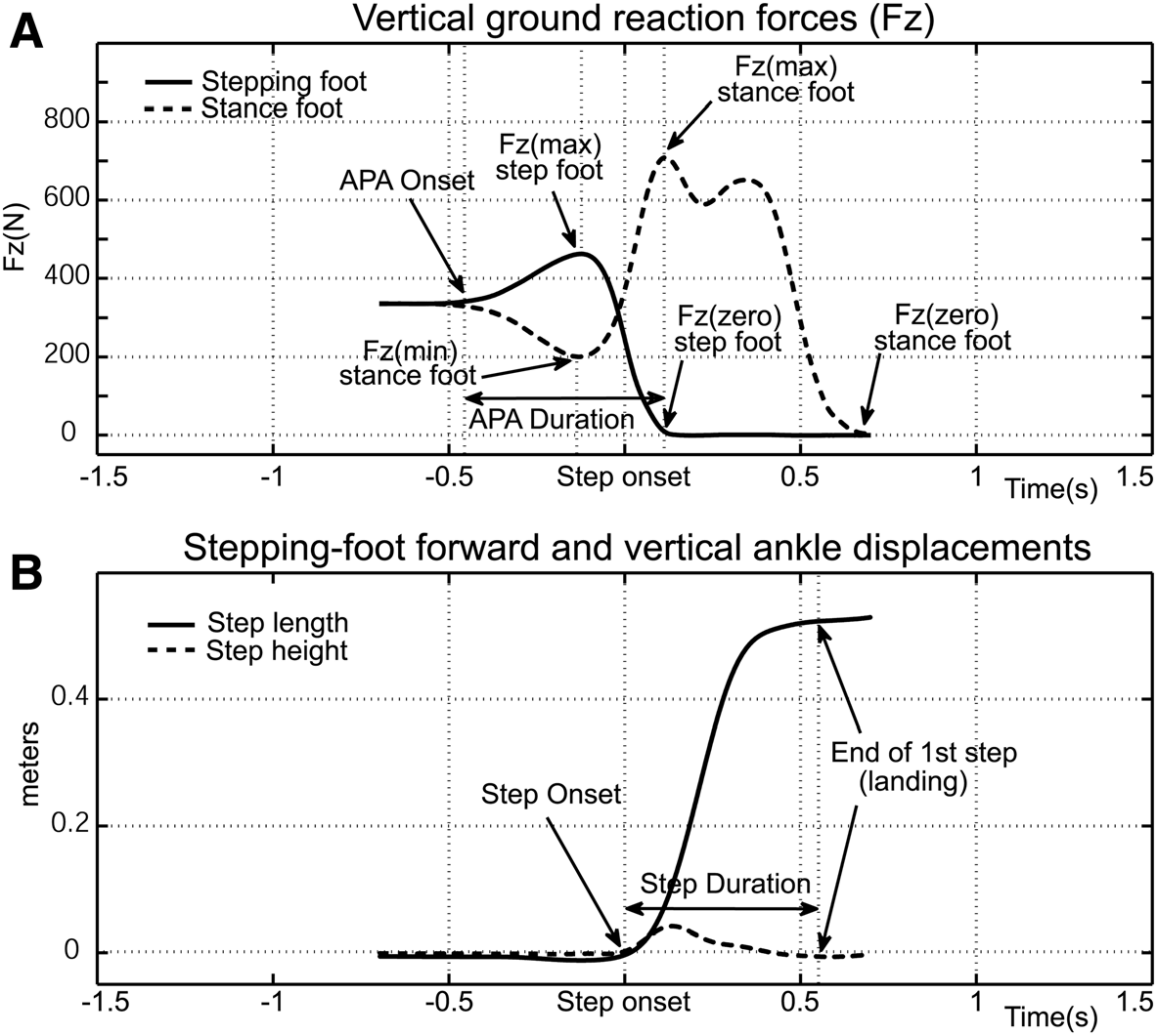
Time series of APA Fz (A) and first step ankle displacement (B) for healthy elderly subjects from APA onset to first step landing.

Vertical forces values (e.g. Fz(max) of the stepping foot, Fz(min) of the stance foot, and Fz(max) of the stance foot) were normalized to subject body weight as measured immediately prior to APA onset (Figure 2A).

APA time intervals were determined for APA onset to Fz(max) of the stepping foot, APA onset to Fz(min) of the stance foot, and Fz(min) to Fz(max) of the stance foot (Figure 2A).

Fz rates (vertical force rates) included: 1) Loading of the stepping foot: the increase in normalized vertical force from APA onset to Fz(max) divided by the time for the interval; 2) Unloading of the stance foot: the decrease in normalized vertical force from APA onset to Fz(min) divided by the time for the interval; and 3) Loading of the stance foot: the increase in normalized vertical force from Fz(min) to Fz(max) divided by the time for the interval.

Time series and their first derivatives were simultaneously plotted in order to facilitate identification of APAs and stepping parameters. Difficulties arose in determining APA and step onsets. The first derivatives (as a function of time) were determined because: 1) it was easier to differentiate when the ankle was either moving or stationary because the simultaneous change from zero m/s to a positive value in both vertical and horizontal ankle-marker velocities reduced the probability of false identification at step onset and landing; and 2) the first derivative for vertical force changes (Fz rate) reduced the chance of falsely identifying timing reference points (e.g. APA onset, Fz(max)) which were difficult to identify during the baseline period (prior to APA onset) because of much larger vertical force changes due to slow postural drift. First derivatives for vertical forces and kinematic data were filtered at 40 Hz using a 2^nd^ order low-pass Butterworth filter with the MATLAB function filtfilt (MathWorks, Natick, Mass.).

Stepping performance was determined by 3-D kinematic analysis of passive reflective markers affixed to the subject’s left and right lateral malleoli. Step onset was defined as the first observable increase in vertical velocity from zero (m/s) (Figure 2B). The first step ended when marker velocity returned to zero with the foot contacting the support surface (Figure 2B). Step length and duration were recorded. Two stepping performance variables are reported: 1) Step speed: step length divided by step duration; and 2) Step length: the horizontal distance traveled by the marker during the first step.

### Procedures

For all testing and training sessions, subjects were instructed to stand naturally with feet comfortably spaced such that their stance leg was positioned on the drop-assist/vibration section of the platform walkway (stimulus was not applied during testing, only during training sessions). From the quiet stance position, subjects took three steps starting with their preferred leg (subject identified) as quickly as they could. Subjects were instructed to initiate stepping at any self-selected time following a verbal ready cue.

Baseline testing occurred just prior to training, post-testing occurred following the six week program, and retention testing occurred at six weeks after the post-test. Subjects were allowed seated rest anytime during the testing procedure. Testing sessions lasted approximately 3 hours.

Prior to each training session, subjects performed a brief, five-minute warm-up consisting of trunk rotation exercises, marching in place, and walking at various paces (comfortable to fast-paced).

Training involved two sessions per week for six weeks. Subjects performed 60 repetitions during each session of the rapid stepping task. They received the PAL stimulus (drop or vibration) beneath the stance foot for every training trial. Decreases in stance-side vertical ground reaction force of 5% (from baseline) triggered the computer-controlled drop or vibration stimulus. Each training session lasted between 45 minutes and 1 hour. To minimize any fatigue effects, subjects were allowed seated rest whenever requested.

### Data analysis

Individual averages for performance variables were calculated as the arithmetic mean across five trials with the exception of two subject in the vibration group and three subjects in the drop group for whom individual averages were determined using 4 trials each. Group averages were calculated as the arithmetic mean across subjects, respectively. Variance was reported as the standard error of the mean.

Statistical significance was determined using group (2) × test session (3) repeated-measures ANOVAs for each of the performance variables. Post-hoc differences were assessed using Tukey-Kramer. Differences were considered significant for p < .05.

## Results

### Time series of vertical forces and kinematic data

In order to provide a formative frame of reference for examining the PD data, Figure 2A shows the averaged vertical ground reaction forces (Fz) for step initiation for 8 healthy elderly subjects during M-L APAs (5 males/3 females age = 73.3 ± 9.1(s.d.) years, data courtesy of M.W. Rogers). Initially, subjects are standing quietly with their weight evenly distributed between both feet. At approximately 0.5 seconds before step onset the Fz starts to increase under the stepping foot (solid line) while simultaneously decreasing beneath the stance foot (dashed line), indicating that the M-L push to achieve single-limb stance through redistribution of the net center of pressure has started. Maximum Fz beneath the stepping foot and minimum Fz beneath the stance foot are attained about 120 ms before step onset. Next, Fz is shifted from the stepping foot to the stance foot establishing single-limb support. The swing phase begins as the stepping foot is lifted vertically (Figure 2B dashed line) and propelled forward (Figure 2B solid line). Note that step onset, defined as the first increase in vertical velocity of the ankle, occurs prior to the stepping-foot Fz reaching zero due to lingering contact of the forefoot with the support surface.

**Figure 3:**
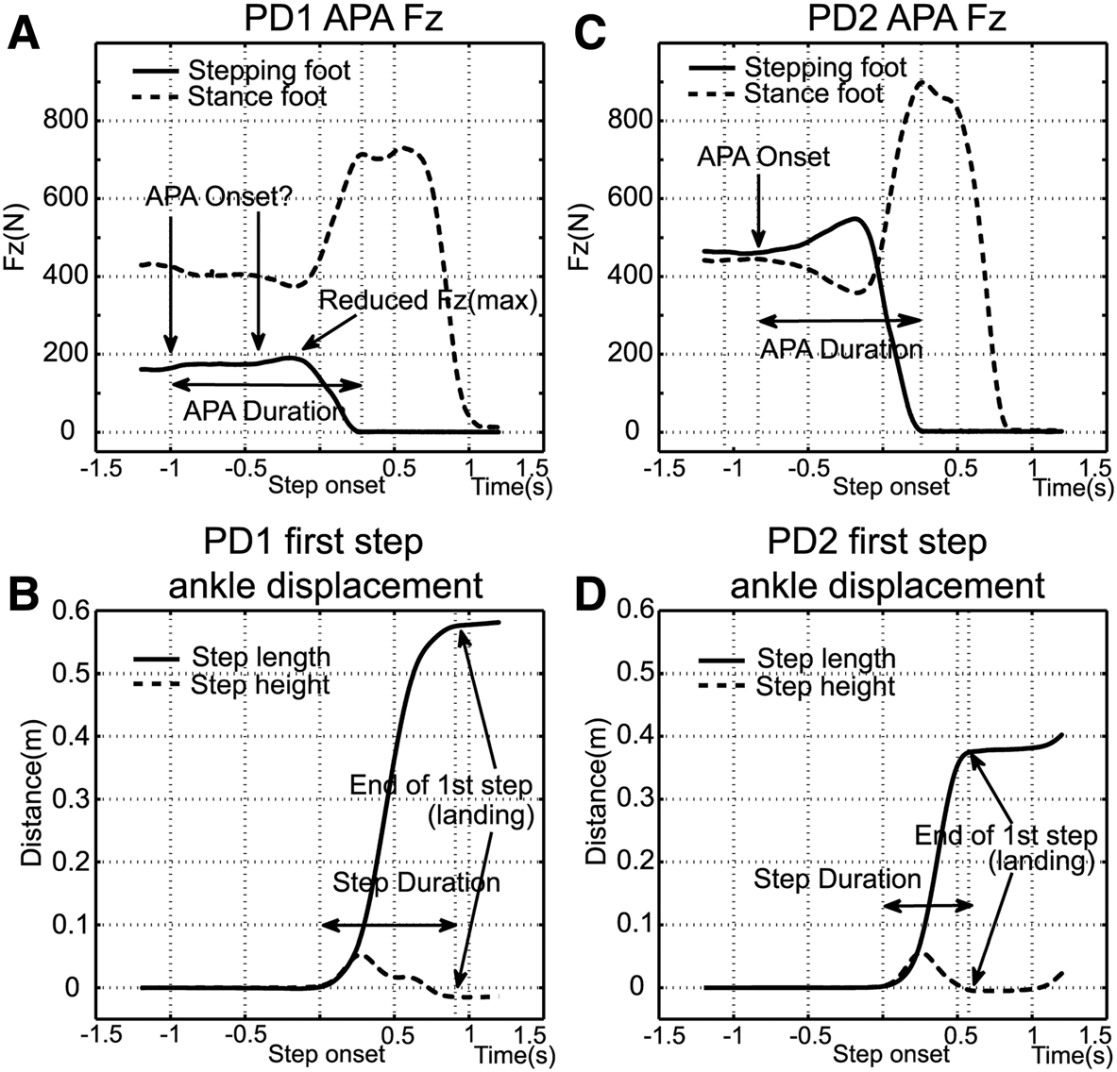
Average time series of APA Fz (A&C) and first step ankle displacement (B&D) for two PD subjects from APA onset to first step landing.

**Figure 4:**
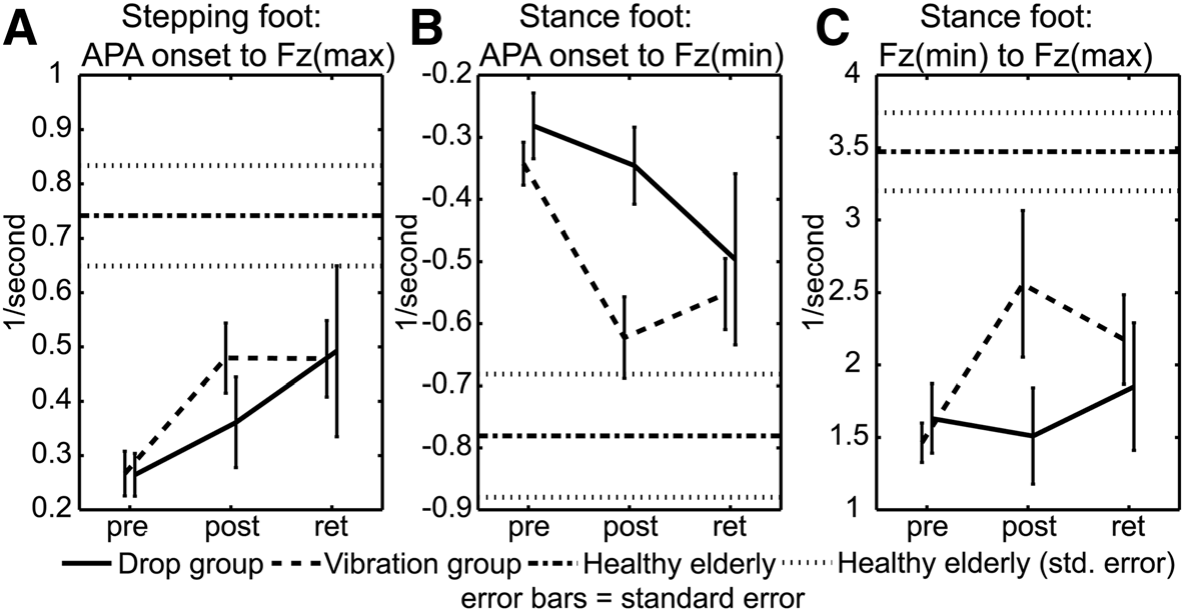
APA Fz loading rates for: A. Stepping foot APA onset to Fz(max); B. Stance foot APA onset to Fz(min); and C. Stance foot Fz(min) to Fz(max).

**Figure 5:**
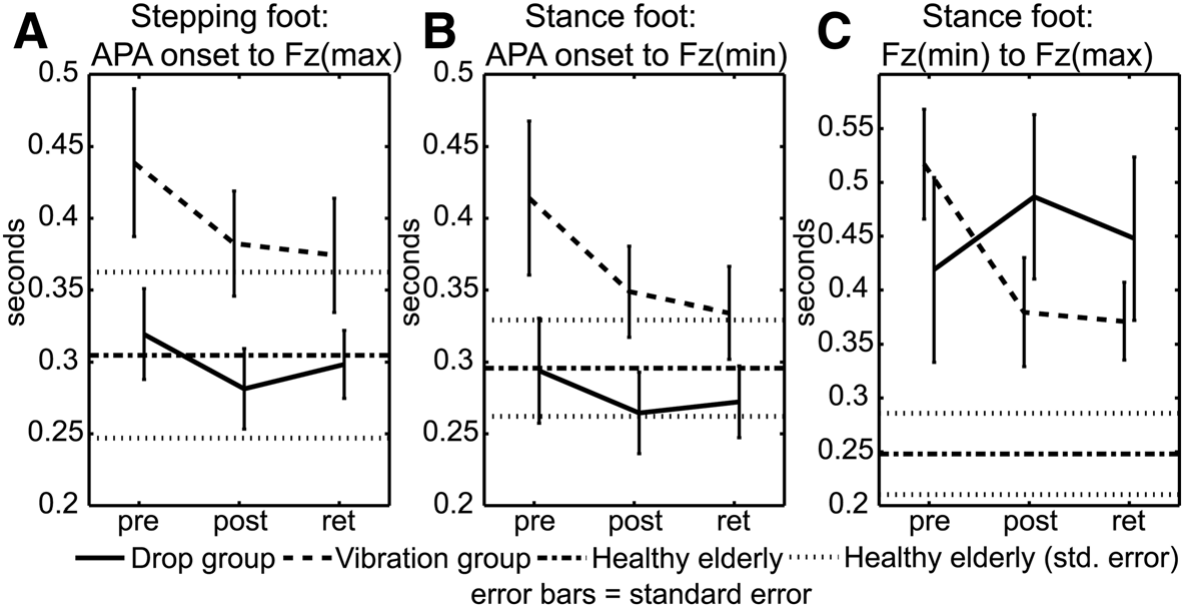
APA time intervals for: A. Stepping foot APA onset to Fz(max); B. Stance foot APA onset to Fz(min); and C. Stance foot Fz(min) to Fz(max).

A selected display of APA and stepping performance in persons with PD can be seen in Figure 3. We show the same Fz measurements for two PD subjects (averages for 5 trials each) in Figures 3A & B (PD1) and 3C & D (PD2) for comparison with the average values for healthy elderly displayed in Figures 2A & B. PD1 (Figure 3A & B) was observed throughout the testing and training regimen as displaying greater step-performance deficiencies. In general the subject displayed pronounced hesitation during step initiation and labored to complete the steps, but particular attention should be paid to several important points: 1) the subject’s pre-step weight distribution favors the stance limb by a significant margin (Figure 3A) while weight for the healthy elderly subjects is evenly distributed between both feet (Figure 2A); 2) APA onset is difficult to identify, i.e. the loading of the stepping foot (solid line) and unloading of the stance foot (dashed line) are barely discernible (Figure 3A) and are significantly less pronounced than the values observed for healthy elderly (Figure 2A); 3) the first significant increase in Fz for the stepping foot and the first significant decrease in Fz for the stance foot occur approximately 1 second before step onset (Figure 3A). By comparison, the time between APA onset and step onset is significantly shorter for the healthy elderly subjects, lasting on average 0.5 seconds (Figure 2A); 4) the rate of stepping-foot loading and stance foot unloading (Figure 3A), i.e. the slopes of the changes in Fz leading up to Fz(max, stepping foot) and Fz(min, stance foot), are small compared to those displayed by the healthy elderly subjects (Figure 2A); 5) first step duration, i.e. step onset to landing, was slightly less than 1 second (Figure 3B). By comparison, the average step duration for the healthy elderly subjects was approximately 0.5 seconds (Figure 2B).

By comparison, PD2 (Figure 3C & D) represented the less-affected end of the performance spectrum. The subject was better able to control their weight distribution, showed very little hesitation, was able to step vigorously, and performed similar to healthy elderly subjects with the following exceptions: 1) vertical peak force differences, i.e. baseline to Fz(max, stepping foot) and Fz(min, stance foot), were reduced (Figure 3C) compared to those of healthy elderly subjects (Figure 2A); 2) the first significant increase in Fz for the stepping foot and the first significant decrease in Fz for the stance foot occurred slightly less than 1 second before step onset (Figure 3C), significantly longer than the average values for healthy elderly of 0.5 seconds (Figure 2A).

In these examples, stepping performance does not differ substantially between healthy elderly and PD subjects. PD1 took the longest step (Figure 3B), while PD2 took the shortest (Figure 3D). The average for the healthy older subjects was between the two PD subjects (Figure 2B). Note that despite the observed performance differences, both PD subjects were subjected to the same inclusion/exclusion criteria and were clinically assessed at a similar level of deficit (H&Y 2.5).

### APAs: vertical force rates

Figure 4A shows a significant test-session (i.e. includes all subjects) stepping-foot main effect for the time interval from APA onset to Fz(max) (p = 0.004). Post-hoc tests revealed that the increase occurred between pre- and retention tests (p(pre,ret) = 0.016), from 0.267 1/s (normalized rate units) to 0.480 1/s.

Figure 4B shows a test-session main effect for the stance foot for the time interval from APA onset to Fz(min) (p = 0.001). Post-hoc tests show that the changes in Fz rate occurred between the pre-test value of −0.314 (units = 1/s) to the post-test value of −0.493 (p(pre,post) = .031). Note that more-negative numbers indicate an increased rate of stance-foot unloading. The retention test value, -0.526 1/s, was also significant (p(pre,ret) = 0.009).

Significant test-session main (p = 0.047) and interaction effects (p = 0.025) for the stance foot Fz(min) to Fz(max), the rate of stance-limb loading, are shown in Figure 4C. The test-session main effect for Fz rate increased from an initial value of 1.543 1/s to 2.025 1/s for the retention test (p(pre,ret) = 0.036). The interaction effect was due to an increase in stance limb loading by the vibration group from 1.465 1/s to 2.560 1/s between pre- and post-tests (p(pre,post) = 0.001) which remained significant through the retention test at 2.177 1/s (p(pre,ret) = 0.024).

### APAs: timing

APA timing was characterized as the incident timing of maximum and minimum Fz values. There were no significant main or interaction effects for stepping-foot APA onset to Fz(max) (Figure 5A) or stance-foot APA onset to Fz(min) (Figure 5B). A significant interaction effect was found for the timing interval defining loading of the stance limb, Fz(min) to Fz(max) (Figure 5C). This interaction effect (p = 0.036) was due to a vibration-group decrease in time duration from 0.517 s to 0.380 s between pre- and post-tests (p(pre-post) = 0.0229) which was maintained at the retention test value of 0.371 s (p(pre-ret) = 0.016).

### APAs: magnitude, timing, and rate changes

It is important to note that the significant changes in Fz rates for early APA loading of the stepping foot (APA onset to Fz(max)) and early unloading of the stance foot (APA onset to Fz(min)) (Figure 4A &4B) occurred due to changes in the magnitude of the APA force and not due to a decrease in the time interval (Figure 5A &5B). By comparison, later during the APA the significant change in loading rate for the stance foot (Fz(min) to Fz(max)) (Figure 4C) occurred due to both an increase in the magnitude of the APA force and a decrease in the time interval (Figure 5C).

### Stepping performance

**Figure 6:**
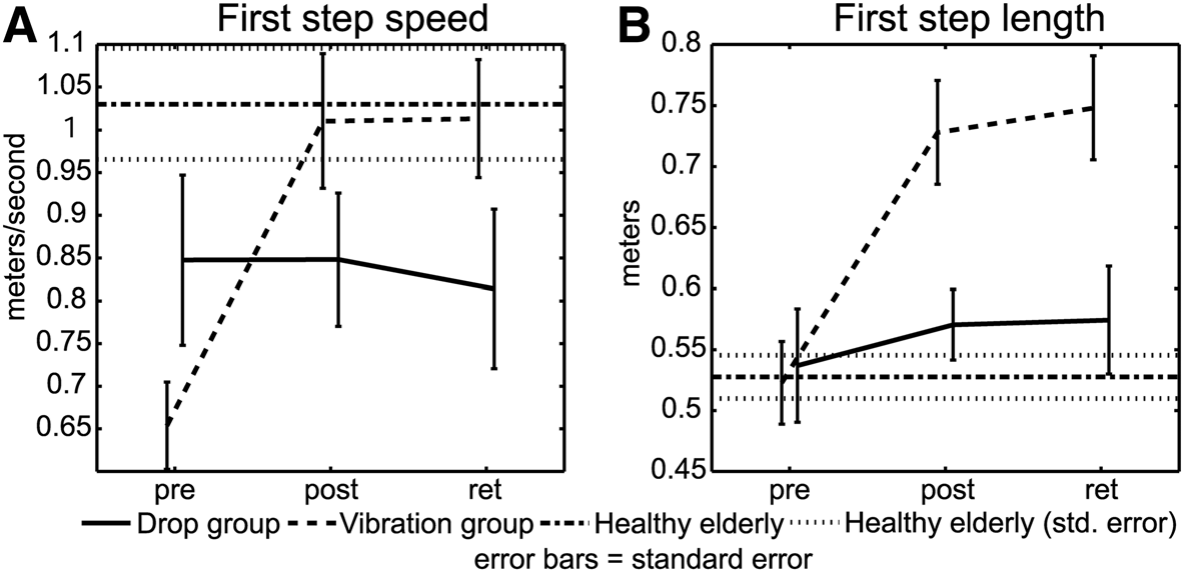
Stepping performance for: A. First step speed; and B. First step length.

Figure 6A shows significant test-session main (p = .0000) and interaction effects (p = .0000) for increases in step speed. Step speed increased from a baseline level of 0.744 m/s to a post-test level of 0.935 m/s (p(pre,post) = 0.001). The increase was retained at 0.920 m/s (p(pre,ret) = 0.003). The interaction effect was the result of increased step speed for the vibration group where step speed increased from an initial value of 0.654 m/s to a post-test value of 1.010 m/s (p(pre,post) = 0.000) and remained higher at 1.013 m/s for the retention test (p(pre,ret) = 0.000). The drop group did not show a significant increase in step speed.

A test-session main effect was observed for increased step length (p = 0.000) (Figure 6B). Test-session step length increased from 0.529 m to 0.654 m (p(pre,post) = 0.002) and was retained at a value of 0.667 m (p(pre,ret) = 0.001). A significant interaction effect was observed due to a vibration group step-length increase (p = .0013). Vibration group step length increased from 0.523 m to 0.728 m (p(pre,post) = 0.000) and increased through retention at 0.748 m (p(pre,ret) = 0.000). Changes in step length were not significant for the drop group.

## Discussion

PD subjects responded positively to the PAL training program. Improvements were observed in several of the APA performance variables for both training groups. Increases in stepping performance were only observed for the vibration training group.

### APAs: force modulation

Previous studies of PAL have shown limited changes in APA force modulation for PD subjects except for increased peak APA amplitudes following acute exposure to the drop stimulus [21]. In contrast, the present study primarily showed significant changes in force modulation with prolonged exposure to training.

During early APA loading of the stepping foot, both groups showed similar improvements in force modulation between pre- and retention tests (Figure 4A). The vibration group showed greater improvement between pre- and post-tests than the drop group, but both groups demonstrated similar performance levels for the retention test. The difference between the groups appears to be due to three drop subjects who failed to show improvement for the post-test, two of whom showed later improvement for the retention test. The reason for this delayed training response is not known.

Similar improvements during the early APA in Fz(min) rate (Figure 4B) were noted for unloading of the stance foot. The post-test difference in group results for stance foot unloading is more pronounced than for loading of the stepping foot. The vibration group clearly shows greater improvement for the post-test while the drop group reaches approximate parity by the retention test. The difference between the two groups is driven by the same three individuals who failed to show improvements for stepping foot loading.

Improvements in late APA force modulation occur for Fz(min) to Fz(max), loading of the stance foot. Stance foot loading between pre- and post-tests is dominated by significant test-session and interaction effects of the vibration group with the drop group showing a delayed training response (Figure 4C). Post-test group differences were driven by four subjects including the three previously mentioned.

Previous experiments employing PAL stimuli have noted deficits in APA vertical force modulation compared to healthy controls [21,28,29]. An important observation of the present study is that, despite the noted deficiencies, force modulation is a very robust training variable.

Whereas previous experimental results [21,29,30] mainly indicated improved APA timing in response to PAL enhancement, the current results indicated that APA force modulation was the most responsive training variable. This occurred despite four key points: 1) The training program contained no strength enhancing exercises; 2) Prior to training, subjects displayed no obvious deficiencies in strength; 3) The drop stimulus provided neuromechanical assistance to lateral weight shift that enhanced APA quickness, as opposed to strength; and 4) Instructions given to subjects emphasized stepping quickly. If anything, the present protocol favored improving performance timing. The fact that improvements in force modulation dominated the findings suggests that, although deficient in PD [21,28-30], APA force control may be a readily adaptable training variable and should be targeted in rehabilitation of posture and locomotion.

### APAs: timing changes

Previous results showed that PD subjects had decreased APA time duration [21,28,29] and earlier first step onset times associated with the acute effects of PAL stimulus exposure [21,28,29]. In contrast, improvements in time-related variables were noted only for the vibration training group for the interval from Fz(min) to Fz(max) of the stance limb (Figure 5C). Increasing the force (or rate of force application) immediately at APA onset by quicker loading of the stepping foot would be an effective way to initiate quick lateral weight shift and achieve the timing improvements observed in previous experiments. However, the current findings pertaining to the stance limb improvements suggest that while rapid APA loading of the stepping limb is necessary to initiate lateral weight shift, controlled unloading and reloading of the stance foot appears to be an equally important variable in enhancing forward locomotion. Furthermore, the fact that training improvements in timing occurred later in the APA phase, expands upon the idea of improving start hesitation delays by providing stimulus enhancement near the time of APA onset [21,28,29]. Thus, it is possible that there may be two responses to PAL training, an acute transient response that is sensitive to the early, time-related characteristics of the PAL stimulus and a more persistent long-term response that is sensitive to stimulus characteristics associated with loading the stance limb and controlling balance during single-limb support.

### Stepping performance

Previously, Mille et al. [28,29] and Rogers et al. [21] demonstrated that PD subjects improve stepping performance with exposure to a facilitative, lateral weight-shift stimulus, implemented either by waist-pulls or dropping the support surface. It was these early results that provided the primary impetus in the design of this experiment. The hypothesis was that mechanical facilitation of a neurally-controlled process affecting posture and locomotion coupling, i.e. APA lateral weight shift, was the reason for the observed improvements in APAs and stepping performance. However, the improvements in stepping performance observed here occurred only for the vibration group where subjects achieved a 55% increase in stepping speed (Figure 6A) and 39% increase in step length (Figure 6B) which were retained at six weeks post-training. The improvements in stepping performance achieved by the vibration group were not expected. The original purpose of the vibration stimulus was to provide a non-mechanical sensory timing cue that occurred at the same relative point during the lateral weight shift as the drop stimulus under the assumption that the drop stimulus may be facilitating achievement of the intended postural state conditions at the onset of lateral weight shift by providing timing as well as mechanical enhancement for the sequential release of the stepping cycle [20]. Since there was no facilitative weight shift assistance provided by the vibration stimulus, training-related improvements were expected to be less than for the drop group. While the reasons behind these improvements are not fully understood, at least three possibilities exist: 1) The training response to the sensory cue provided by the vibration stimulus may be more robust than the response to the mechanosensory cue provided by the drop stimulus; 2) Subjects in the vibration group may have simply responded to training better than the drop group subjects; and 3) Since there were no restrictions against outside-of-training activities, It’s possible that the vibration subjects pursued more challenging activities outside of training or during the untrained retention period.

By comparison, the drop training group did not improve their stepping performance with longer-term training. This contrasts with our previous findings focused on acute effects of PAL showing immediate improvements in stepping performance [21,28,29]. Hence, the present results indicated that the drop stimulus enhanced APA performance, but not stepping.

The possibility of confounding factors to treatment outcomes was explored by looking at associations between stepping performance and other factors. However, no significant relationships were found for duration of PD, gender, medication regimen, level of frailty, or any other subjective observations.

## Conclusions

It appears as though the PAL stimulus is acutely effective at improving APA quickness and immediate stepping performance [21,28,29], but that sensitivity to the quickness-enhancing effects is supplanted by improved force modulation following prolonged stimulus exposure. Nevertheless, the results of this study encourage further applications of the PAL training approach in the context of other functionally relevant whole-body posture and locomotion sequencing tasks that become progressively disordered with advancing PD, e.g. rising from a chair, sustaining ongoing gait, turning while walking, passing through doorways, and reaching while standing.

## Abbreviations

PDs: Persons with Parkinson’s disease; PD: Parkinson’s disease; APA: Anticipatory postural adjustments; FOG: Freezing of gait; M-L: Medial-lateral; A-P: Anterior-posterior; CNS: Central nervous system; PAL: Posture assisted locomotion; UPDRS: Unified Parkinson’s Disease Rating Scale; Fz: Vertical force; Fz(max)/Fz(min): Maximum/minimum value of vertical force; H&Y: Hoehn and Yahr disability score for PDs.

## Competing interests

The authors declare that they have no competing interests.

## Authors’ contributions

RC was principle author and responsible for data analysis. MP was responsible for analyzing clinical data, screening subjects and data collection. LS was clinical neurologist specializing in movement disorders who was responsible for subject screening and advising. MH was responsible for data collection and analysis. KM was responsible for data collection and analysis. CM was instrumental in experimental design. M-LM was instrumental in experimental design. TS was clinical neurologist specializing in movement disorders who was responsible for subject screening and advising. JZ was responsible for data collection and analysis. MWR developed experimental paradigm, designed testing equipment, performed data collection, and was contributing author. All authors read and approved the final manuscript.
